# Acute appendicitis in overweight patients: the role of preoperative imaging

**DOI:** 10.1186/s13037-016-0102-0

**Published:** 2016-05-17

**Authors:** Marc-Olivier Sauvain, Sandra Tschirky, Michael A. Patak, Pierre-Alain Clavien, Dieter Hahnloser, Markus K. Muller

**Affiliations:** Department of Visceral and Transplantation Surgery, University Hospital Zurich, Zurich, Switzerland; Department of Visceral Surgery, University Hospital CHUV, Switzerland Rue du Bugnon 46, CH- 1011 Lausanne, Switzerland; Radiology, Klinik Hirslanden, Zurich, Switzerland; Cantonal Hospital Frauenfeld, Frauenfeld, Switzerland

**Keywords:** BMI, Appendicitis, CT scan, Appendectomy, Ultrasound

## Abstract

**Background:**

The diagnosis of acute appendicitis in overweight patients is challenging due to the limited value of the clinical examination. The benefits of ultrasonography and abdominal CT have been studied in the general population, but there is limited data regarding their use in overweight and obese patients with suspected appendicitis. This study analyzes the role of preoperative radiological modalities in overweight patients with suspected appendicitis.

**Methods:**

Retrospective analysis of a prospectively acquired database including 705 patients operated for suspected acute appendicitis. Patients were divided into two groups according to their BMI (BMI ≥25 kg/m^2^ (*n* = 242) and BMI <25 kg/m^2^ (*n* = 463)). The use of preoperative radiological modalities, laboratory findings and outcome parameters were analyzed.

**Results:**

Ultrasonography was the preferred radiological assessment in our cohort (68 % in BMI <25 kg/m and 52.4 % in BMI ≥25 kg/m^2^). However, it was non-conclusive in 42 % of overweight as compared to 6 % in patients with a BMI < 25 (*p* < 0.0001). This difference was particularly obvious between female patients (8 % of non-conclusive US for BMI <25 kg/m^2^ vs 52 % for BMI ≥25 kg/m^2^, *p* < 0.0001). Significantly more CT scans were performed in overweight patients (37 % vs. 20 %; p <0.0001). The accuracy of CT did not differ according to BMI (85 % vs. 88 %; *p* = 0.76). Preoperative radiological imaging did not significantly delay surgery. Laparoscopy was the preferred approach for both groups (98.2 % vs 98.7 %, *P* = 0.86) with an overall conversion rate of 4 %. The overall rate of negative appendectomy was 10 %.

**Conclusions:**

The role of ultrasonography in patients with BMI ≥25 kg/m^2^ with suspected acute appendicitis is questionable due to its high rate of non-conclusive findings. Therefore, abdominal CT scans should be preferred to investigate suspected appendicitis in overweight patient if clinical findings are not conclusive.

## Background

In Switzerland, 51 % of men and 32 % of women present a Body Mass Index (BMI) ≥25 kg/m^2^ and the percentage of obese patients (BMI ≥30 kg/m^2^) nearly doubled over the last 20 years [1]. Overweight patients presenting with an acute abdomen are a real challenge for clinicians as larger volumes of intra-abdominal and subcutaneous fat can affect the accuracy of physical examination [2–4]. The benefits of ultrasonography and abdominal CT scan in the diagnosis of acute appendicitis have been studied in the general population [5–14]. However, there is limited data regarding their role in overweight patients with suspected acute appendicitis [4].

The aim of this study was to analyze the role of preoperative radiological modalities in overweight patients with suspected appendicitis.

## Methods

We present a retrospective analysis of a prospectively acquired database of all consecutive patients operated for suspected appendicitis at a teaching hospital between January 2005 and March 2011. The data are part of a quality assessment project held by the “Verein outcome” (http://www.vzk-qualitaetsbericht.ch/). This non-profit organization was founded by the State of Zurich to monitor the health care quality of various interventions in public hospitals. Included were all patients older than 16 years operated for suspected appendicitis.

The “Verein outcome” did not give recommendations on any aspect of the medical process. The indication for surgery was made by the attending surgeon on call based on the patient’s personal history, clinical status, imaging and laboratory findings. There was no standardized diagnostic algorithm or predefined surgical strategy. Indication for radiological investigations was at the discretion of the surgeon.

The following variables were collected: age, gender, BMI, laboratory parameters (white blood count, C-reactive protein), histological findings, preoperative radiological modalities when used, operation method, delay between admission and surgical incision. This time difference between admission and surgery was used to calculate the door to scalpel time. Surgical specimens were analyzed by a trained pathologist and classified as no appendicitis or appendicitis.

The data collection was anonymized and collected by the outcome association in accordance with the local ethical comity.

The term non-conclusive was used when the radiological report could neither confirm nor exclude an appendicitis. We determined the accuracy of the radiological modality by comparing to histological findings and the radiological reports.

All parametric data are presented as median values with interquartile rages (IQR). Comparison of data between groups of patients was performed using the Chi square test for discrete variables and the Mann- Whitney-U-test/Student’s t-Test for parametric data. A *p*-value <0.05 was considered significant.

## Results

### Patient demographics

A total of 805 consecutive patients were diagnosed for suspected acute appendicitis and underwent appendectomy. One hundred patients were excluded for the following reasons: 99 had no BMI in the database and one patient was transferred to another hospital before surgery. In total, 705 patients with complete data were included in the final analysis (Table 1). The median age was 33 (25–43) years. Three hundred eighty-eight patients (55 %) were male and 317 were female (45 %).

**Table 1:** Patients demographics

	BMI <25 kg/m^2^	BMI ≥25 kg/m^2^	*p*-value
N (%)	463 (66 %)	242 (34 %)	-
BMI kg/m^2^ (median)	22 (20–23)	28 (26–31)	≤0.0001
Age (years)	29 (23–39)	37 (30–51)	≤ 0.0001
Gender (male/female)	231/232	157/85	≤ 0.0001
Leucocytes count (10^3^ G/l)	13.2 (10.6–15.8)	13.1 (10.6–16.0)	0.52
CRP (mg/l)	21 (5.0–70.0)	27.0 (8.9–95.5)	0.02
Door to scalpel time (min)	485 (335–720)	508 (354–757)	0.63

All results presented in median (interquartile range)

Patients were divided in two groups according to their BMI with a cut off value of 25 kg/m^2^, corresponding to the overweight value defined by the WHO [15]. Four hundred sixty-three patients (66 %) had a BMI <25 kg/m^2^ and 242 (34 %) were overweight or obese. The BMI <25 group had a median BMI of 22 kg/m^2^ (20–23) whereas the BMI ≥25 group had a median BMI of 28 kg/m^2^ (26–31). Patients within the former group were younger (29 years (23–38) vs. 37 years (30–51); *p* < 0.0001). The overweight group was composed of 157 men (65 %) and 85 females (35 %), whereas in the Group with a BMI < 25 kg/m2 the gender distribution was even (231male vs 232 female). The laboratory parameters indicative for inflammation did not show any clinically relevant differences between both groups (Table 1).

### Pre-operative imaging

Pre-operative imaging was performed in 624 patients (89 %). Eighty-one patients (11 %) were operated without preoperative imaging, among which 28 patients were females (35 %) and 53 were males (65 %; *p* = 0.6). Only 44 females (51 %) in the BMI ≥25 group were operated without CT scan compared to 190 (81 %) in the other group (*p* < 0.0001) (Table 2). Ultrasonography and CT-scan were used as single radiological modality in 443 (63 %) and 101 (14 %) patients, respectively.

**Table 2:** Pre-operative imaging according to BMI

	BMI < 25 kg/m^2^	BMI ≥ 25 kg/m^2^	*p*-value
No radiological examination	55 (11.9 %)	26 (10.7 %)	0.75
Sonography only (%)	316 (68.2 %)	127 (52.4 %)	<0.0001
CT only (%)	41 (8.8 %)	60 (24.7 %)	<0.0001
Both: sonography & CT (%)	51 (11 %)	29 (12 %)	0.25
No CT in female	190 (81 %)	44 (51 %)	<0.0001

### Pre-operative imaging by BMI

Three hundred sixteen (68 %) BMI <25 patients and 127 (52 %) BMI ≥25 patients were assessed by ultrasonography (*p* < 0.0001). In the latter group ultrasonography was significantly more often used for male than female (72 % vs. 28 % respectively; *p* < 0.0001). Sixty patients (25 %) underwent CT in the BMI ≥25 group, whereas 41 (9 %) patients in the BMI <25 group (*p* < 0.0001). In 80 (11 %) patients, ultrasonography and computed tomography were performed at the same frequency within both groups (11 % vs. 12 %, *p* = 0.25) (Table 2).

### Accuracy of the radiological modality

If ultrasonography was performed the diagnosis of appendicitis was confirmed histologically in 61 % in the BMI <25 group (Table 3). The percentage of non-conclusive ultrasonography increased from 6 % for BMI <25 up to 42 % for BMI ≥25 patients (*p* < 0.0001). The rate of non-conclusive ultrasonography increased especially for overweight female patients (52 % vs 8 % for BMI <25, respectively *p* < 0.0001).

**Table 3:** Accuracy of imaging modality

	BMI < 25 kg/m^2^	BMI ≥ 25 kg/m^2^	*p*-value
Sonography accuracy	223 (61 %)	83 (53 %)	0.12
Non-conclusive sonography			
- for female	21 (6 %)	66 (42 %)	<0.0001
	15 (8 %)	25 (52 %)	<0.0001
CT accuracy			
- for female	81 (88 %)	76 (85 %)	0.76
	37 (82 %)	33 (80 %)	0.89
Non-conclusive CT			
- for female	10 (11 %)	5 (6 %)	0.31
	6 (13 %)	4 (4 %)	0.78

The accuracy of CT did not differ according to BMI (88 % in BMI <25 group vs. 85 % in BMI ≥25 group; *p* = 0.76). Regardless of the BMI, CT scan was more accurate for male patients (91 and 90 % in BMI <25 and BMI ≥25) than for female patients (82 and 80 %, respectively).

### Operative data and histological findings

Laparoscopic appendectomy was performed on 694 (98 %) patients with a conversion rate of 4 % (*n* = 27) (Table 4). The surgical approach did not differ significantly between both groups (98.2 and 98.7 % laparoscopic appendectomy (*p* = 0.22) for BMI < 25 and ≥25, respectively). The conversion rate was higher in the BMI <25 group than the BMI ≥25 group without reaching significance (5 %, *n* = 21 vs. 2.6 %, *n* = 6; *p* = 0.25).

**Table 4:** Operative procedure and histology

	BMI < 25 kg/m^2^	BMI ≥ 25 kg/m^2^	*p*-value
Type of surgery			
- open (%)	8 (1.7 %)	3 (1.2 %)	0.86
- laparoscopy (%)	434 (98.2 %)	233 (98.7 %)	0.13
- conversion (%)	21 (5 %)	6 (2.6 %)	0.25
Negative appendicectomy	49 (10.5 %)	21 (8.7 %)	0.50
- for female	33 (7.1 %)	10 (4.1 %)	0.16

The overall rate of negative appendectomy was 10 %. The rate of negative appendectomy did not differ between overweight and non-overweight patients (Table 4). In patients without any preoperative imaging appendicitis was confirmed by pathology in 94 % of the cases independently of both BMI (91 and 100 % for BMI <25 and ≥25 respectively, *p* = 0.27) and gender (female 90 and 96 % male, *p* = 0.33) (data not shown).

### Door to scalpel time

The median door to scalpel time in our cohort is 495 min (340-740) and does not significantly differ between groups (Table 1). Moreover, in both groups the preoperative imaging did not delay surgery (Table 5). It took no longer if ultrasonography (500 min.; *p* = 0.87), CT scan (490 min.;*p* = 0.71) or both investigation were performed (513 min.; *p* = 0.59) compared to no imaging 455 min.

**Table 5:** Door to scalpel time according imaging modality

	Imaging	No imaging	*p*-value
Sonography (min)	500 (335–720)	455 (335–737)	0.87
CT (min)	490 (345–783)	455 (335–737)	0.71
Sonography & CT (min)	513 (364–742)	455 (335–737)	0.59

All results presented in median (interquartile range)

## Discussion

This study demonstrated that the BMI influenced the choice of primary preoperative imaging in patients with suspected appendicitis. Patients with a BMI ≥25 kg/m^2^ had nearly twice as many CT scans performed as patient with a BMI <25 kg/m^2^ (37 % vs. 20 % respectively, *p* < 0.0001). In addition, the percentage of non-conclusive ultrasonography increased from 6 to 42 % in patients with a BMI ≥25 kg/m^2^, with an accuracy rate of only 53 %. Performing radiological imaging did not delay surgery in this study.

Fifty seven percent of the world’s adult population are expected to be overweight or obese in 2030 [16]. These rapid and dramatic physical changes will have an impact on the clinical evaluation of patients, also for common problems such as appendicitis. A careful history and a meticulous physical examination remain the cornerstone in diagnosis of an acute appendicitis. However, the physical examination of obese patients is challenging and provides less reliable information increasing the need of additional exams [17].

Numerous studies have demonstrated the superiority of CT scan over ultrasonography to detect acute appendicitis in the general population [8–14], but not in overweight patients. In studies from the Netherlands and Belgium (prevalence BMI ≥25 kg/m^2^of 47.8 %, in 2008) ultrasonography was recommended as first-line preoperative modality for reasons of costs and availability, even if computed tomography was more accurate. Portmann et al. advised to add computed tomography only when ultrasonography was negative or non-conclusive [14]. However, it is known that ultrasonography is less accurate in obese patients [13]. This is in accordance with our data showing that overweight patients had more non-conclusive ultrasonography. Therefore, the cost/effectiveness of ultrasonography as the first line modality for overweight patients with suspected appendicitis should be analyzed in the future. Even more so as our data suggested that patients with a BMI >25 kg/m^2^ would benefit from an abdominal CT rather than ultrasonography if clinical examination is non-conclusive. In the United States, a country with a high prevalence of overweighed population, computed tomography is well established as the preoperative modality of choice (70 % use of CT) [16].

If the history and clinical examination clearly suggests an acute appendicitis, additional imaging is not necessary. All twenty-six overweight patients who did not undergo any preoperative radiological examination in this study had an acute appendicitis confirmed by pathology (zero negative appendectomy). This might be surprising and one might expect a higher negative appendectomy rate. Especially, as the literature suggests that the negative appendectomy rate had decreased since the introduction of preoperative radiological modality [9, 18, 19]. This is even more true for overweight patients [4]. One reason could be that the mean BMI of these 26 patients was 29 kg/m2 which could allow a better clinical evaluation than in obese patients. We can also speculate that those patients had clear signs of peritonitis as the mean CRP was 86 mg/l which was nearly three times more than the median for the group of patients with radiological imaging.

Despite the benefits of CT regarding the diagnostic accuracy of appendicitis, the radiation exposure for the patient is not insignificant. The percentage of radiation induced cancer by increased used of CT is estimated to be at 2 % in the U.S.[20]. This must be taken into account especially in overweight children and woman in childbearing age. Improved CT protocols have been published and low dose CT for all patients might be a solution in the future [21, 22]. The use of MRI could become an alternative in the future but its costs and its poor availability are not yet compatible with an emergency setting. In this study, we showed that the time from admission to surgery was not delayed by the use of preoperative imaging. If easy accessible and in the case of non-conclusive clinical findings an abdominal CT should be performed in the overweight patients.

As mentioned earlier, computed tomography leads to higher costs compared to ultrasonography. However, considering the percentage of inconclusive ultrasonography in overweight patients, the number of false positive diagnoses would increase without CT and the number of negative appendectomies would rise. This again is associated with higher costs for health care system [23]_._ In addition, the risk of perioperative mortality could increase, when an inconclusive acute and severe intra-abdominal process is mistakenly considered as an appendicitis [24]. Appendectomy for appendicitis was the treatment of choice over the past 120 years and is currently being debated. Recent studies indicated that acute non perforated appendicitis could be successfully treated conservatively without operation [25, 26]. In the future, we will need to differentiate uncomplicated from perforated appendicitis and tailor the treatment accordingly. Computed tomography will most likely gain further importance, given the endemic nature of overweight patients and the change of treatment of acute appendicitis.

The costs of the health care are increasing dramatically worldwide and this trend will not decline in the future [27]. One challenge of future clinical practice lies in rethinking established preoperative evaluations and to adjust them for overweight patients in order to improve the cost/benefits ratio.

The sub-group analysis of female patient with a BMI ≥25 kg/m^2^ is particularly interesting as the US was non-conclusive in more than 50 % of the cases. This contrast with the 8 % non-conclusive US for female with BMI <25 kg/m^2^. The complex anatomy of the women coupled with the low lead of US in obese patients should prompt the clinicians to perform rather a CT scan if the clinical evaluation of these patients is not obvious.

This study has several limitations. First, the indication to perform radiological imaging was not standardized. We assumed that preoperative radiological assessment was only used, when the clinical presentation was not clear. Secondly, we do not know the outcome of patients who had a negative radiology examination and did not undergo appendectomy. Finally, BMI was unknown for 12.3 % of patients (they were therefore excluded from the present analysis). However, the negative appendectomy rate in this group was comparable to the rest of our cohort (data not shown).

## Conclusion

The role of ultrasonography in patients with BMI ≥25 kg/m^2^ with suspected acute appendicitis is questionable due to its high rate of non-conclusive findings. Therefore, abdominal CT scans should be preferred to investigate suspected appendicitis in overweight patient if clinical findings are not conclusive.

### Ethics approval

The data collection was anonymized and collected by the outcome association in accordance with the local ethical comity.

### Source of funding

None.

## Abbreviation

BMI: body mass index
CT: computer tomography
IQR: interquartile Range
US: ultrasonography
